# Circumferential strain predicts major adverse cardiac events independent of myocardial perfusion in asymptomatic aortic stenosis

**DOI:** 10.1186/1532-429X-14-S1-P90

**Published:** 2012-02-01

**Authors:** Sacha Bull, Joseph J Suttie, Harry Willis, Jubin Joseph, Jane M Francis, Theodoros Karamitsos, Stefan Neubauer, Saul Myerson

**Affiliations:** 1 OCMR, Oxford, UK

## Background

Asymptomatic aortic stenosis (AS) remains a challenging clinical entity characterised by adverse left ventricular remodelling, microvascular dysfunction and sudden cardiac death. Previous studies have shown reduced myocardial strain in patients with asymptomatic aortic stenosis, however the relationship between impaired myocardial strain, ischaemia and major adverse cardiac events (MACE) remains poorly understood. We therefore assessed myocardial strain and myocardial perfusion reserve index (MPRI) in these patients and followed patients prospectively.

## Methods

96 consecutive patients (73 male) underwent CMR scanning on a 1.5T scanner with a 32-channel coil. Of these, 45 underwent adenosine stress perfusion scanning. The remaining 51 patients had medical contraindications for perfusion imaging or refused consent. LV mass and MPRI were analysed and late gadolinium enhancement imaging (LGE) was performed using standard protocols, and scored by two independent operators blinded to other CMR results. Patients were then followed prospectively for a mean of 1.5 ±0.8 years and the predictors of MACE determined.

## Results

**Figure 1:**
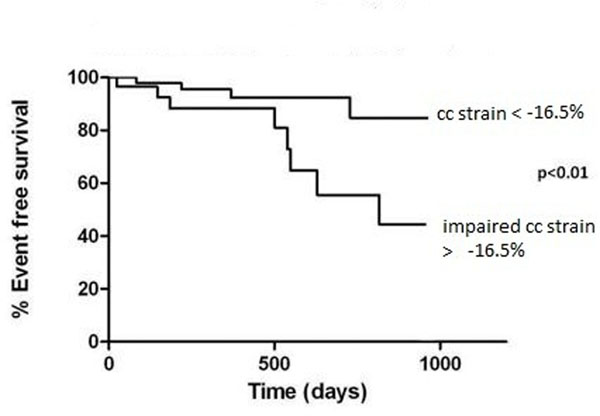
Survival in patients with AS and impaired LV circumferential strain (cc).

19 of 96 patients suffered a MACE: 1 death, 12 valve replacements, 1 atrial fibrillation, 1 pacemaker insertion, and 4 unplanned hospitalisations with cardiac symptoms. Patients with MACE had significantly impaired mid ventricular peak systolic circumferential strain compared to those without MACE (-14.8±3% vs -17.9±3% respectively, p<0.001, figure 1), and also had impaired diastolic torsion rate (-25±10os-1vs -34±15os-1, p=0.04). The MACE event rate in the patient group with reduced circumferential strain (> -16.5) was 37%, compared to 16 % in patients with strain <-16.5. The relative risk of MACE in those with impaired strain was 2.2. There were no significant differences in longitudinal strain (-11.5±3% vs -12.0±3%, p=0.5); presence of LGE (p=0.3), BNP levels (18.7±27pmol/L vs 18±31pmol/L, p=0.9), LVEF (69±10% vs 70±9%, p=0.74) or mass index (90±23gm-2 vs 83±22 gm-2, p=0.23). In those with perfusion data, the mean MPRI was 1.3± 0.4. Those with lower perfusion reserve (below the mean) had higher transvalvular velocities (3.7±0.6m/s) compared to those with MPRI>1.3 (3.0±0.6m/s); p=0.002). There was no correlation between reduced strain and reduction in MPRI, though numbers were more limited. There was also no significant difference in MPRI values between those with normal or reduced strain, and between those with/without MACE.

## Conclusions

There is a 2.2-fold increase in MACE in patients with AS and reduced circumferential strain compared to those with normal myocardial strain. Myocardial perfusion reserve (a measure of microvascular dysfunction) was not related to strain or to MACE.

## Funding

British Heart Foundation, Heart Research UK, Oxford Biomedical Research Centre, National Institute for Health Research.

